# Impact of a Nurse Practitioner–Led Diabetes Program on Barriers to CGM Use in a Federally Qualified Health Center After Medicaid Expansion

**DOI:** 10.1155/jdr/5724236

**Published:** 2025-11-06

**Authors:** Ligaya Docena Scarlett, Walter Solorzano, Katayoun Khoshbin, Giuliana Perini Villanueva, Kathyana Santiago Mangual, Marielle Tavares, Cynthia Santana, Bryan Escobar, Joseph Borrell, Beatrice Brumley, Tannaz Moin, Estelle Everett

**Affiliations:** ^1^Venice Family Clinic, Los Angeles, California, USA; ^2^Department of Medicine, University of California, Los Angeles, Los Angeles, California, USA; ^3^Division of General Internal Medicine & Health Services Research, University of California, Los Angeles, Los Angeles, California, USA; ^4^David Geffen School of Medicine, University of California, Los Angeles, Los Angeles, California, USA; ^5^Division of Endocrinology, Diabetes, & Metabolism, University of California, Los Angeles, Los Angeles, California, USA; ^6^School of Nursing, University of California, Los Angeles, Los Angeles, California, USA; ^7^Department of Medicine, VA Greater Los Angeles Healthcare System, Los Angeles, California, USA

**Keywords:** CGM, continuous glucose monitoring, federally qualified healthcare centers, FQHC

## Abstract

**Objective:**

Continuous glucose monitors (CGMs) enhance diabetes management, but disparities exist, particularly among underserved populations in federally qualified health centers (FQHCs). In 2022, a California Medicaid policy change expanded CGM coverage, providing an opportunity to better evaluate barriers to CGM use within primary care in an FQHC.

**Methods:**

We used 2022–2023 electronic health record (EHR) data to identify adults with diabetes managed on insulin within a nurse practitioner–led diabetes program in primary care. Patients were categorized as current, former, or never CGM users. We used summary statistics, chi-squared, and Bartlett's tests to assess unadjusted group differences and multivariate logistic regression to identify factors associated with former or never use. All patients were invited to complete a survey on CGM facilitators and barriers.

**Results:**

Among 275 eligible patients, 109 (40%) were current CGM users, 31 (11%) former users, and 135 (49%) never users. Discussions on CGM occurred in 45% of never users, who were more likely to have non-Medicaid insurance, fewer than five clinic visits (OR 3.69, 95% CI: 1.94–6.99), and a lower baseline A1C (OR 0.67, 95% CI: 0.52–0.86). No demographic or clinical factors were associated with former CGM use. Among survey respondents (*n* = 124), the desire to reduce finger-pricks motivated CGM use, while device burden and inconvenience contributed to discontinuation or refusal.

**Conclusions:**

Medicaid policy expansion reduced major structural barriers to CGM use, yet some patient-related barriers persisted. Team-based care models integrating health educators and advanced practice providers can successfully support CGM access and sustained use in underserved populations.

## 1. Introduction

Continuous glucose monitoring (CGM) is an invaluable tool that significantly improves health outcomes in people with diabetes (PWD). The benefits of CGM have been well documented in both Type 1 and Type 2 diabetes and include reductions in hemoglobin A1C, fewer hospitalizations, improved patient satisfaction, and decreased diabetes-related distress [1–4]. Despite these advantages, studies consistently report inequities in CGM use among racial and ethnic groups, individuals with lower socioeconomic status, and those with public insurance [5–7]. CGM use is particularly low in federally qualified health centers (FQHCs) [5–8]. In 2024, a study with 36,385 FQHC patients found that only 1.3% used CGMs [5]. Low CGM utilization in this setting represents a missed opportunity, as PWD receiving care in these centers often experience poorer glycemic control [9]. Prior research exploring the drivers of gaps in CGM use have identified multiple barriers to CGM initiation [6, 10–16]. This includes disparities in the discussion and prescribing of CGMs, as well as patients being lost to follow-up after conversations about initiating CGMs [6]. Inadequate insurance coverage for CGM has been found to be one of the largest contributors to CGM disparities [6, 10–16].

In January 2022, California's Medicaid program (Medi-Cal) expanded its coverage to include CGM use for all PWD using insulin, providing coverage under a united pharmacy benefit instead of durable medical equipment (DME) [17]. During this transition, Medi-Cal temporarily suspended prior authorization requirements for 9 months to facilitate access [18]. This shift in Medi-Cal policy presented a unique opportunity to examine CGM utilization in FQHCs, where the majority of patients are Medi-Cal recipients.

Venice Family Clinic (VFC) is a multisite FQHC in Los Angeles County that operates a Diabetes Management Program (DMP) embedded within its primary care clinics. The DMP is staffed by nurse practitioners (NPs) who are also certified diabetes care and education specialists (CDCES), alongside health educators (HEs), who frequently support CGM use. While numerous studies have highlighted disparities and barriers to CGM uptake, few have described real-world care models aimed at addressing these challenges—particularly within FQHCs—or examined the residual barriers that persist when major structural obstacles, such as insurance coverage, have been removed. This is the first known study to examine facilitators and barriers to CGM use within an NP-led diabetes program at an FQHC following the 2022 Medi-Cal policy change.

## 2. Methods

### 2.1. Diabetes Management Program

The DMP is a primary care-based program that largely supports patients with A1C levels greater than 9%, who are either referred by their providers or identified through population health outreach initiatives. Patients are seen every 3–6 weeks by NPs who are paired with a diabetes HE at their clinic site. HEs are nonlicensed staff trained by the clinical team to provide diabetes support and education. NPs provide medication management and insulin dose adjustments, while HEs provide additional support at the point of care and between visits, including patient education, case management, and diabetes device training. All NPs and HEs conduct discussions regarding CGM use and provide education in the patient's preferred language.

HEs facilitate point-of-care CGM initiation, enabling patients to begin using a CGM device immediately following their medical visit with the NP. This was possible due to the availability of on-site pharmacies or by manufacturer-provided starter kits to ensure that all patients received the same benefit of immediate initiation. HEs guide patients through the step-by-step process of CGM use, which often includes assisting with the creation of email accounts or resetting passwords to enable application downloads on patients' smartphones. They support patients during the initial sensor application, and continued hands-on support is offered through follow-up visits every 10–14 days, during which HEs assist with sensor changes until patients are comfortable independently changing the device.

### 2.2. Chart Review

We reviewed electronic health records (EHRs) of patients who had been seen at least twice by one of the diabetes NPs between the study period of January 2022 and June 2023. Eligible patients were aged 18 years or older with insulin-managed diabetes (documented diagnosis of Type 1 diabetes [T1D] or Type 2 diabetes [T2D] managed on basal insulin or multiple daily injections [MDIs] ± other agents). We stratified patients into three categories of CGM utilization: current users, former users, and never users. Current users were defined as those who were CGM users at the start of the study period or were initiated on CGM within the study period and continued its use through the end. Former users were those who used CGM but discontinued use before the end of the study period. Never users were patients who did not use CGM at any point during the study period.

We used EHR data to collect baseline demographic data and clinical characteristics, including A1C, insulin regimens, diagnoses of diabetes-related complications, and the number of visits with the diabetes NP during the study period. Among former users, manual chart review was also performed to assess reasons for CGM discontinuation.

For patients categorized as never users, clinical notes were reviewed to determine whether CGM was discussed during any clinical encounters. Keyword searches were conducted within the medical record using terms such as “CGM,” “sensor,” “Libre,” and “Dexcom” to identify documentation of such discussions. When a discussion was found, we evaluated whether a CGM prescription was entered during the same visit. If CGM was discussed but not prescribed, we further reviewed the chart for documentation as to why the patient did not initiate CGM.

### 2.3. Survey

To further explore patients' perspectives on facilitators and barriers to CGM use, we invited individuals identified in the chart review to participate in one-on-one telephone surveys, which were conducted between November 2023 and February 2025. Survey items inquired about reasons for CGM use or nonuse across the three utilization groups: current users, former users, and never users. Current users were asked about their primary reason for starting a CGM and motivations for continued use. Former users were asked about their initial reasons for CGM use and the factors that led to discontinuation. Never users were asked whether CGM had ever been recommended by a clinician and, if so, their reasons for declining. Additionally, never users were asked during the survey whether they would consider using CGM if it were made available to them and, if not, to provide their rationale for continued nonuse. Surveys were administered by research staff in the patient's preferred language, and verbal consent was obtained. Participants received a $50 gift card upon the completion of the survey.

### 2.4. Statistical Analysis

Summary statistics (means with standard deviation and relative proportions) were used to describe demographic and baseline characteristics' differences between the three CGM user groups (e.g., current, former, and never). Between-group differences were tested using the chi-squared test for categorical variables and Bartlett's test for equal variances for continuous variables. We performed a multivariate adjusted logistic regression model among patients who ever used CGM to determine which patient and clinical factors were associated with discontinuing CGM. Similarly, a multivariate adjusted logistic regression model was performed among all participants to evaluate patient and clinical factors associated with never using a CGM. Survey responses were summarized and reported descriptively.

## 3. Results

### 3.1. Chart Review

Among the 669 patients who had clinic visits with the diabetes NPs during the study period, 275 patients met our inclusion criteria. Most were female (57%), aged 50–64 years (46%), and were predominantly Hispanic ethnicity (85%) with a primary language of Spanish (71%) (Table 1). The majority had T2D (93%) and were insured by Medi-Cal (63%). Our study sample included 40% current CGM users (*N* = 109), 11% former CGM users (*N* = 31), and 49% never users (*N* = 135).

#### 3.1.1. Current CGM Users

Approximately 78% used intermittently scanned CGMs (isCGM) and 28% real-time CGMs (rtCGMs). While most current users had T2D (88%), patients with T1D had a significantly higher proportion of current CGM users compared to former users and never users (*p* = 0.029). CGM users were more likely to be on basal/bolus insulin ± other agents (*p* = 0.009) and had higher rates of Medi-Cal coverage (84%) (*p* < 0.001).

#### 3.1.2. Former CGM Users

All former users were previously on an isCGM. Reasons for discontinuing CGM per chart review included patient preference, body aches, sensors falling off, Bluetooth connection issues, and false low readings. A multivariate logistic regression model was performed to predict former CGM use compared to current CGM use. Using an isCGM perfectly predicted former CGM use and was excluded from the model. No other significant associations were found between former CGM use and demographics (age, sex, race/ethnicity, language, and insurance) or clinical factors (DM type, number of complications, current regimen, number of visits, or baseline A1C).

#### 3.1.3. Never CGM Users

Among CGM never users, provider discussions about CGM were documented in 44.4% of cases, though reasons for declining were often missing (53.3%). In multivariate analysis, insurance status was a strong predictor of never using CGM. Compared to Medi-Cal, patients with private insurance (OR 13.80, 95% CI 1.50–127.40; *p* = 0.021), Medicare (OR 2.55, 95% CI 1.09–6.00; *p* = 0.031), or no insurance (OR 20.33, 95% CI 6.87–60.11; *p* < 0.001) had significantly higher odds of never use. Fewer DMP visits (2–5 vs. > 5) were also associated with higher odds of never use (OR 3.69, 95% CI 1.94–6.99; *p* < 0.001). Additionally, each 1% increase in baseline A1C was associated with a 33% reduction in the odds of being a never user (OR 0.67, 95% CI 0.52–0.86; *p* = 0.001), and patients not on basal/bolus insulin regimens were nearly twice as likely to be never users (OR 1.92, 95% CI 1.04–3.59; *p* = 0.039).

### 3.2. Survey

We invited all 275 patients identified in the chart review to complete a survey, and 124 (45%) agreed to participate. Among these, 55% were either current users, 15% former users, and 30% had never used a CGM.

**Figure 1:**
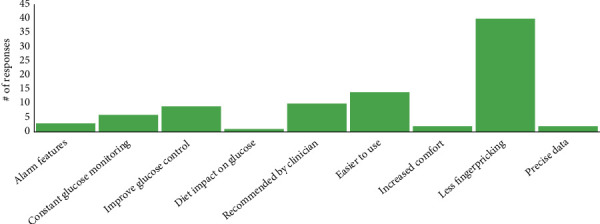
Top reason for starting CGM.

Among current CGM users, top reasons for CGM use included reducing finger-pricking (45%), ease of use (15%), clinician recommendations (11%), and improved glucose control (10%) (Figure 1). Among former users, the most common reasons for discontinuing were frequent alarms (50%), unwanted public attention from alarms (50%), insurance issues (33.3%), skin reactions (33.3%), and excessive worry about glucose levels (33.3%) (Table 2).

Among never users, 55.6% had never received a provider recommendation (Table 2). Those who declined a CGM despite a recommendation cited concerns about wearing a device (56.3%), the hassle of continuous wear (56.3%), fear of device malfunction (43.8%), and discomfort with frequent glucose monitoring (31.3%). When asked about openness to future CGM use if made available, 47.2% were open to it, while 52.8% declined. Among those not interested, key reasons included preferring glucometers (94.7%), disliking wearable devices (78.9%), and finding CGMs inconvenient (83.3%). Patient quotes illustrating their rationale are presented in Table 3.

## 4. Discussion

In the DMP, 51% of patients using insulin used CGM, which is substantially higher than the 1.3% national FQHC average [5]. This success highlights the combined impact of Medi-Cal policy changes and a care model specifically designed to overcome barriers to CGM access and sustained use. The VFC model featuring proactive CGM prescriptions, same-day device initiation, and ongoing support resulted in the majority of patients beginning and sustaining CGM use after the California Medicaid policy change. Discontinuation rates were low, with 78% of patients maintaining CGM use throughout the study period.

Limited CGM knowledge and time constraints were found to be major barriers to discussing CGM use with patients in a national survey of over 1300 primary care clinicians [19, 20]. The VFC model addressed these challenges by leveraging NPs and HE to promote CGM adoption and champion its use among primary care patients. Although provider bias has been shown to negatively impact CGM utilization among adults with diabetes [6], we found no significant difference in CGM use by age, race, or language, and almost half of the patients who never initiated CGM had documented discussions with their NP about CGM use.

The DMP also reduced the likelihood that patients would be lost to follow-up after CGM discussions by leveraging HEs to provide point-of-care CGM training and placement. Prior research shows that training support staff to perform CGM placement increased CGM use in a safety-net endocrinology clinic [21]; our findings extend this evidence by highlighting the critical role of this approach in facilitating successful CGM implementation within primary care at an FQHC.

Despite these successes, challenges remained. Insurance status remained a major barrier, particularly for those without Medi-Cal insurance. In our regression model, uninsured patients were more likely to have never used CGM, and among those who never had a documented discussion with their provider about CGM, half were uninsured (data not shown). Patients with private insurance or Medicare also had higher odds of never using CGM compared to those with Medi-Cal. This may reflect greater out-of-pocket costs, such as copays, for privately insured and Medicare patients. Additionally, because CGMs are classified as DME under Medicare [22], patients may face further approval and administrative barriers to accessing devices.

Usability concerns impacted CGM initiation; patients commonly citing fear of wearing a device and potential pain as reasons for declining to use CGM. Despite advances in newer CGM technology, we see that patients continue to cite concerns about device obtrusiveness and fear of discomfort as major factors influencing patients' decisions to avoid CGM [10, 23]. Opportunities for peer-led CGM education sessions by community members using CGM devices or providing patients with the opportunity to see and interact with a CGM device during initial discussions, such as observing a trial device worn by a staff member or clinician, may help demystify the technology and increase openness to trialing CGM use in these patients.

Usability challenges also drove CGM discontinuation. In our chart review data, all patients who discontinued CGM were using an isCGM system. This may reflect limitations of these devices, such as the need for manual scanning and the lack of real-time alerts, potentially reducing perceived usefulness and contributing to discontinuation. As isCGM are now phasing out of clinical care, rtCGMs address many of these limitations, which may support greater sustained use. Former CGM users in our survey cited factors including alarm fatigue, adhesive or skin irritation, and anxiety associated with constant awareness of glucose levels as reasons to discontinue CGM. These findings are consistent with existing literature in other patient populations and highlight patient-level barriers contributing to lower rates of sustained CGM use [6, 10–16, 23]. This suggests the importance of support staff, not only during CGM initiation but also for ongoing support for troubleshooting CGM-related issues to improve long-term engagement and comfort with CGM technology.

This study has several limitations. First, data can be incomplete or inconsistently documented in EHRs. To help address this limitation, we also invited all participants to complete surveys. Almost half of the invited participants completed the survey (124 of 275; 45%), and the survey population closely reflected the diversity of the larger patient population of the clinic. Nevertheless, the potential for selection bias remains [15]; however, the consistency between survey responses and chart review findings strengthens the credibility of our results.

This study was conducted at an FQHC in Los Angeles County serving primarily Spanish-speaking PWD, over 80% of whom identified as Hispanic. Therefore, findings may not be generalizable to more culturally or linguistically diverse FQHCs. All the NPs and HEs in the DMP were fluent in Spanish. As a result, findings may differ in populations where there is less language concordance between patients and providers. The temporary suspension of prior authorization requirements during the first 9 months of the study period reduced administrative barriers to CGM access, which may not reflect conditions in current clinical practices. Lastly, although patients were not seen in endocrinology practices, they were managed in a well-established model of care by primary care NPs/CDCESs with advanced diabetes training [24], which may not be widely available across all FQHCs. We did not collect data on CGM use among primary care patients not seen by the diabetes NPs, limiting our ability to compare outcomes between those enrolled in the DMP and those who were not. Nonetheless, our findings suggest that this model is a promising strategy to facilitate the use of diabetes technology in the FQHC setting.

## 5. Conclusions

In primary care FQHC settings, gaps in diabetes technology use have long persisted. Although the Medi-Cal policy change addressed a primary barrier to CGM initiation, our study found that additional barriers related to perceived usability and wearability challenges remained, even in the context of structural and clinical support to facilitate CGM use. Insurance coverage, proactive and knowledgeable clinicians, and a team-based model of care that includes specialty-trained advanced practice providers and HEs can successfully facilitate CGM use. By leveraging these strategies, FQHCs can overcome long-standing barriers and promote more equitable use of diabetes technology. Continued research and efforts are essential to assess the impact of coverage policies and real-world care models on CGM use, ensuring that all patients, particularly those in underserved communities, have access to effective diabetes management tools.

## Figures and Tables

**Table 1:** Baseline characteristics from chart review.

**CGM user status**					
**Variable**	**Current (** **n** = 109**)**	**Former (** **n** = 31**)**	**Never (** **n** = 135** )**	**Total (** **n** = 275**)**	**p** **value**
Age					0.776
< 50	23%	16%	23%	22%	
50–64	49%	48%	43%	46%	
65+	28%	36%	34%	32%	
Birth sex					0.830
Female	60%	55%	56%	57%	
Male	40%	45%	44%	43%	
Race					0.464
Hispanic	81%	81%	89%	85%	
White	10%	13%	6%	8%	
Black/African American	6%	3%	4%	5%	
Asian	1%	3%	1%	1%	
Unknown/unreported	2%	0%	0%	1%	
Primary language					0.120
English	37%	29%	22%	28%	
Spanish	62%	71%	77%	71%	
Other	1%	0%	1%	1%	
Baseline insurance type					< 0.001
Medi-Cal	84%	74%	43%	63%	
Medicare	13%	16%	23%	18%	
No insurance	2%	10%	29%	16%	
Private	1%	0%	5%	3%	
DM type					0.029
1	12%	3%	4%	7%	
2	88%	97%	96%	93%	
Baseline medication regimen					0.009
U500+	5%	0%	0%	2%	
Basal/bolus+	31%	26%	20%	25%	
Basal/bolus only	17%	16%	11%	14%	
NPH/R+	0%	3%	1%	1%	
Basal+	43%	39%	60%	51%	
Noninsulin drugs only	4%	16%	8%	7%	
# of DMP visits					0.001
2–5 visits	20%	32%	44%	33%	
> 5 visits	80%	68%	56%	67%	
Baseline A1C					0.046
< 7%	2%	6%	4%	4%	
7%–7.9%	13%	7%	18%	14%	
8%–8.9%	23%	7%	28%	24%	
9%–9.9%	22%	19%	13%	18%	
10% or greater	40%	61%	37%	40%	
CGM type					—
rTCGM	28%	0%	—	11%	
isCGM	72%	100%	—	40%	
# of DM complications, mean ± SE	1.28 ± 0.11	1.38 ± 0.22	1.21 ± 0.09	1.26 ± 0.67	0.489
Baseline A1C %, mean ± SD	10.02 ± 2.27	10.28 ± 2.19	9.71 ± 2.41	9.91 ± 2.33	0.724

**Table 2:** Barriers to CGM uptake and use.

**Never users**	
*Has your healthcare provider ever recommended that you start CGM?*	% (*N* = 36)
Yes	44.4
No	55.6
*If yes, what reasons made you decide not to use CGM?*	% (*N* = 16)
Unable to attend necessary training/visits with diabetes educator to obtain CGM	18.8
Not enough support from your diabetes care team in using devices	12.5
Not enough information in your preferred language	12.5
Insurance coverage or cost of supplies	12.5
Hassle of wearing devices all the time	56.3
Concerns about having a device on your body	56.3
Worries about other noticing the device on your body	31.3
Nervous that the CGM might not work	43.8
Do not want to have too much information about your glucose levels	31.3
Do not want to take more time from your day to manage diabetes	25
Too busy to learn how to use a CGM	12.5
*Would you consider using CGM now if made available to you?*	% (*N* = 36)
Yes	47.2
No	52.8
*What reasons make you still decline to use a CGM?*	% (*N* = 18)
Concerned that this type of device would be outside of your budget	27.8
Hassle of wearing a CGM all of the time	83.3
You do not like the thought of having a device on your body	78.9
Worries about what others will think of you because the device may be visible to them	33.33
The alarm notifications would be disruptive or bring too much attention in public	55.6
Concerns about the accuracy of this type of device	38.9
It seems too complicated	61.1
The device would take more time from your day to manage diabetes	38.9
You feel more comfortable checking sugar with a glucometer because you are more familiar doing it that way	94.7
**Former CGM users**	
*If you formerly used a CGM, what reasons made you stop wearing it?*	% (*N* = 18)
The sensor readings cannot be trusted	11.1
CGM takes too much time to use	16.7
CGM was too difficult to use	22.2
CGM was not helpful	16.7
CGM was painful to wear	27.8
Did not like having to wear CGM all the time	27.8
CGM caused too much worry about glucose	33.3
It was too hard to understand CGM information and features	16.7
Did not like having CGM on your body or the way it looked on your body	22.2
The adhesive on the CGM caused skin reactions	33.3
Too many alarm notifications	50
Alarm notifications brought too much attention in public	50
Issues with insurance coverage	33.3
Worried about what others thought of you while wearing the CGM	16.7
People noticed the CGM and asked questions about it	29.4
Not enough support from your diabetes care team in using the CGM	0

**Table 3:** Patient reasons for CGM status.

**Reasons for starting CGM**	**Reasons for stopping CGM**	**Reasons for never using CGM**
“…too many painful finger pokes for many years”	“I could not tolerate sensor. I could feel generalized body aches after placement”	“I have been preoccupied with my son's surgery”
“It is more practical to check blood sugar in public and at all times”	“Sensor kept falling off. I was worried it would not stay on due to sweating too much”	“It feels unnatural to use an apparatus. If I fall on it, will it cause more pain?”
“My provider who I trust recommended it as it would only be 1 poke every 2 weeks and easier management”	“I had problems connecting to Bluetooth and experienced false lows”	“It would cause me a lot of anxiety to think about monitoring my levels so often”
“My provider told me this would be easier”	“My insurance no longer covered CGM”	“I do not want sensor to get in the way of my work”
“I would react too quickly to Novolog sending me straight to hypoglycemia. I liked the idea that the CGM could warn me ahead of time”	“I did not like that the sensor was constantly getting knocked off”	“Wearing sensor would put me in a bad mood”
“I could no longer get any blood from my fingers when pricking myself”	“The sensor was irritating my arm”	“The alarms would feel too startling for me”
		“Wearing CGM would be too stressful”
		“I have sensory issues and do not want anything attached to my body”

## Data Availability

The data that support the findings of this study are available on request from the corresponding author. The data are not publicly available due to privacy or ethical restrictions.
